# Metabolic pathways and genes identified by RNA-seq analysis of barley near-isogenic lines differing by allelic state of the *Black lemma and pericarp* (*Blp*) gene

**DOI:** 10.1186/s12870-017-1124-1

**Published:** 2017-11-14

**Authors:** Anastasiya Y. Glagoleva, Nikolay A. Shmakov, Olesya Y. Shoeva, Gennady V. Vasiliev, Natalia V. Shatskaya, Andreas Börner, Dmitry A. Afonnikov, Elena K. Khlestkina

**Affiliations:** 1 Institute of Cytology and Genetics SB RAS, Novosibirsk, Russia; 2 Novosibirsk State University, Novosibirsk, Russia; 3 Leibniz Institute of Plant Genetics and Crop Plant Research (IPK), Corrensstr. 3, D-06466 Gatersleben, Stadt Seeland Germany

**Keywords:** Barley, Near-isogenic lines, Phytomelanin, Black lemma, Black pericarp, RNA-seq, Differential expression

## Abstract

**Background:**

Some plant species have ‘melanin-like’ black seed pigmentation. However, the chemical and genetic nature of this ‘melanin-like’ black pigment have not yet been fully explored due to its complex structure and ability to withstand almost all solvents. Nevertheless, identification of genetic networks participating in trait formation is key to understanding metabolic processes involved in the expression of ‘melanin-like’ black seed pigmentation. The aim of the current study was to identify differentially expressed genes (DEGs) in barley near-isogenic lines (NILs) differing by allelic state of the *Blp (black lemma and pericarp*) locus.

**Results:**

RNA-seq analysis of six libraries (three replicates for each line) was performed. A total of 957 genome fragments had statistically significant changes in expression levels between lines BLP and BW, with 632 fragments having increased expression levels in line BLP and 325 genome fragments having decreased expression. Among identified DEGs, 191 genes were recognized as participating in known pathways. Among these were metabolic pathways including ‘suberin monomer biosynthesis’, ‘diterpene phytoalexins precursors biosynthesis’, ‘cutin biosynthesis’, ‘cuticular wax biosynthesis’, and ‘phenylpropanoid biosynthesis, initial reactions’. Differential expression was confirmed by real-time PCR analysis of selected genes.

**Conclusions:**

Metabolic pathways and genes presumably associated with black lemma and pericarp colour as well as *Blp*-associated resistance to oxidative stress and pathogens, were revealed. We suggest that the black pigmentation of lemmas and pericarps is related to increased level of phenolic compounds and their oxidation. The effect of functional *Blp* on the synthesis of ferulic acid and other phenolic compounds can explain the increased antioxidant capacity and biotic and abiotic stress tolerance of black-grained cereals. Their drought tolerance and resistance to diseases affecting the spike may also be related to cuticular wax biosynthesis. In addition, upregulated synthesis of phytoalexins, suberin and universal stress protein (USP) in lemmas and pericarps of the *Blp* carriers may contribute to their increased disease resistance. Further description of the DEGs haplotypes and study of their association with physiological characteristics may be useful for future application in barley pre-breeding.

**Electronic supplementary material:**

The online version of this article (doi: 10.1186/s12870-017-1124-1) contains supplementary material, which is available to authorized users.

## Background

Grain colour is one of the stable taxonomic characters used for the description and classification of barley (*Hordeum vulgare* L., *2n* = *2×* = 14, HH) accessions. Barley grain at maturity may have white, yellow, purple, blue, or black pigmentation. In white grains, the pigments are absent, whereas two different classes of compounds determine the other types of colours. Yellow, purple, and blue are caused by a diverse subgroup of flavonoid compounds, synthesized in different grain tissues [1, 2]. Unlike the pigmentations caused by flavonoids, studies of black coloration are very scant from biochemical and molecular genetic points of view.

Black coloration is caused by ‘melanin-like’ pigments, which are groups of high molecular weight irregular polymers, arising in the course of oxidation and polymerization of phenolic compounds [3]. Because of their complex structure and resistance to almost all solvents, their chemical nature and the metabolic pathways leading to these black pigments have not yet been determined [4].

In barley, the ‘melanin-like’ pigments accumulate in the lemma and pericarp of grains during the later stages of growth, slightly before maturation of the spike [1, 5]. This trait is controlled by the monogenically inherited *Blp1* (black lemma and pericarp 1) locus that has been mapped to chromosome 1HL [6, 7]. Three dominant alleles of the *Blp1* locus conferring intensity of grain pigmentation have been reported: *Blp1.b* (*B*), *Blp1.mb* (*B* ^*mb*^), and *Blp1.g* (*B* ^*g*^) determine extreme black, medium black, and light black or grey colours, respectively [5, 8, 9].

Barley landraces with black grains were found in southwestern Asia, and sparsely in Tibet and China [10]. In Syria, the black-grained locally adopted landrace ‘Arabi Aswad’ was grown on approximately 75% of the barley growing area, particularly in the driest part of the country, unlike the white-seeded ‘Arabi Abiad’ landrace which was grown in more favourable conditions [11–13]. Compared to white-seeded varieties, black-seeded barley types are considered to be more drought tolerant, more vigorous in early stages of growth, more cold-tolerant, more prostrate, taller and faster-maturing [11, 12].

In addition to better viability under abiotic stress conditions, black-grained barley demonstrate higher resistance to fungal infection. FHB screening showed that among black-grained accessions, 20% were resistant, while among non-coloured accessions only 5% of genotypes were found to be resistant [14, 15]. Barley recombinant inbred lines (RILs) with black grains demonstrated lower Fusarium head blight incidence and lower accumulation of mycotoxin deoxynivalenol than yellow RILs, at least at some disease pressure levels [16]. Increased resistance to *Fusarium* disease has also been reported in oat genotypes having dark-coloured floral glumes in comparison with light-coloured ones [17].

Some studies of the phytochemical composition of barley grains with black colour have revealed that real ‘melanin-like’ black pigments may be present simultaneously with anthocyanins and other related co-pigments that contribute to the total phenol content [18, 19].

Comparative analysis of phenolic profiles and transcription of flavonoid biosynthesis pathway genes in black- and white-grained barley near-isogenic lines (NILs) demonstrated that none of the key flavonoid biosynthesis genes were upregulated in the black NIL, whereas in the presence of a dominant *Blp1* allele, the total amount of phenolic compounds was enhanced in the black-grained line in comparison with the white-grained one [20]. These data suggest that neither coloured (proanthocyanidins, anthocyanins, phlobaphenes) nor uncoloured flavonoids participate in ‘melanin-like’ pigment biosynthesis, and instead, other classes of phenolic compounds are involved.

In the current study, to reveal genetic networks (and hence metabolic pathways) participating in formation of the ‘melanin-like’ black pigments, differentially expressed genes (DEGs) in black- vs white-grained NILs were determined by RNA-seq.

## Results

Six pooled libraries containing a total of 23 million short reads were produced as raw sequence data. After filtering, 2.49 million (15%) reads were removed from libraries before further analysis. Alignment of the libraries to the *H. vulgare* reference genome resulted in an average of 40% reads being mapped for each library. Metrics of library preprocessing and mapping are shown in Table 1.

**Table 1:** Metrics of libraries preprocessing and mapping

	Raw reads, mln	Clean reads, mln	Mean length	Mapped reads (%)
BW1	1,77	1,27	175	37,4
BW2	3,74	3,55	211	32,1
BW3	5,25	4,96	215	46,4
BLP1	3,58	3,22	192	34,9
BLP2	4,71	4,37	165	39,9
BLP3	4,07	3,58	152	41,2

**Fig. 1:**
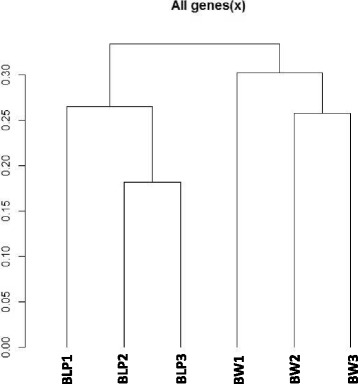
Clustering of mapped libraries based on RPKM values for the transcripts (Additional file 1)

Among all genome fragments, 22,976 were identified as having non-zero expression levels. The fragments, their locations, annotations and respective RPKM values are listed in Additional file 1 (Additional file 1, Table ‘Genes’). These fragments include genes listed in genome assembly annotations as well as genome fragments for which expression has been detected by the Cufflinks pipeline and are further referred to as ‘transcripts’. RPKM values for the transcripts were used for library clustering (Fig. 1). Libraries BLP (2) and BLP (3) are highly similar in terms of gene expression, as are the BW (2) and BW (3) libraries. BLP (1) differs somewhat from the other two libraries belonging to the BLP line. Library BW (1) is further separated from the two other libraries belonging to the Bowman line (Fig. 1). Nevertheless, two clusters can be distinguished on the map, each containing all libraries of one of the two biological lines.

**Fig. 2:**
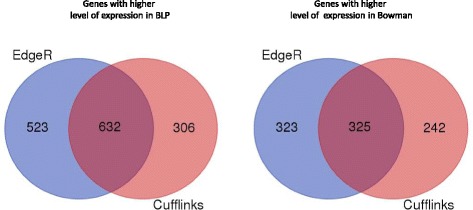
Number of differently expressed genes (DEGs) revealed by Cufflinks and EdgeR

EdgeR detected 648 genes with lower and 1155 genes with higher expression levels in line BLP (Additional file 1, Table ‘EdgeR’), whereas Cufflinks detected 567 and 938 genes with lower and higher levels of expression in line BLP, respectively (Additional file 1, Table ‘Cufflinks’). In the list of genes mutually identified by both tools, 325 genes with decreased and 632 with increased expression levels in BLP were detected (Fig. 2; Additional file 1, Tables ‘UpReg DEGs Cufflinks & EdgeR’, ‘DownReg DEGs Cufflinks & EdgeR’). Thus, 957 transcripts with differential expression were detected in total, comprising 4.1% of all transcripts expressed in either of the biological lines.

PlantCyc database (BarleyCyc division) annotations include 196 differentially expressed genes, identified by Cufflinks and EdgeR, as participating in known BarleyCyc pathways (Additional file 2). The five pathways with the highest DEG PPS scores are ‘superpathway of pyrimidine deoxyribonucleotides *de novo* biosynthesis’, ‘UTP and CTP dephosphorylation II’, ‘flavonoid biosynthesis (in equisetum)’, ‘flavonol biosynthesis’ and ‘UTP and CTP dephosphorylation I’. Pathways with high numbers of DEGs include ‘suberin monomer biosynthesis’, ‘cutin biosynthesis’, ‘phenylpropanoid biosynthesis, initial reactions’, ‘superpathway of scopolin and esculin biosynthesis’ and ‘eugenol and isoeugenol biosynthesis’. Among additional pathways potentially related to the differing phenotypes of the NILs were ‘cuticular wax biosynthesis’ and ‘diterpene phytoalexins precursors biosynthesis’.

Using Blastp, homologies among Affymetrix Barley Genome Array sequences were found for 508 upregulated and 270 downregulated transcripts in BLP. Gene Ontology enrichment analysis showed that the GO term ‘fatty acid biosynthesis process’ is significantly (FDR = 7,5·10^−4^) enriched in transcripts with higher expression in BLP (Additional file 3), while cellular component GO terms ‘nucleoplasm’ (FDR = 0,03) and ‘chloroplast thylakoid’ (FDR = 0,029) and molecular function GO terms ‘transporter activity’ (FDR = 3,4·10^−3^) and ‘transcription factor activity’ (FDR = 5,5·10^−3^) were significantly overrepresented among transcripts with lower expression in line BLP (Additional files 4-5).

### qPCR differential expression verification

**Fig. 3:**
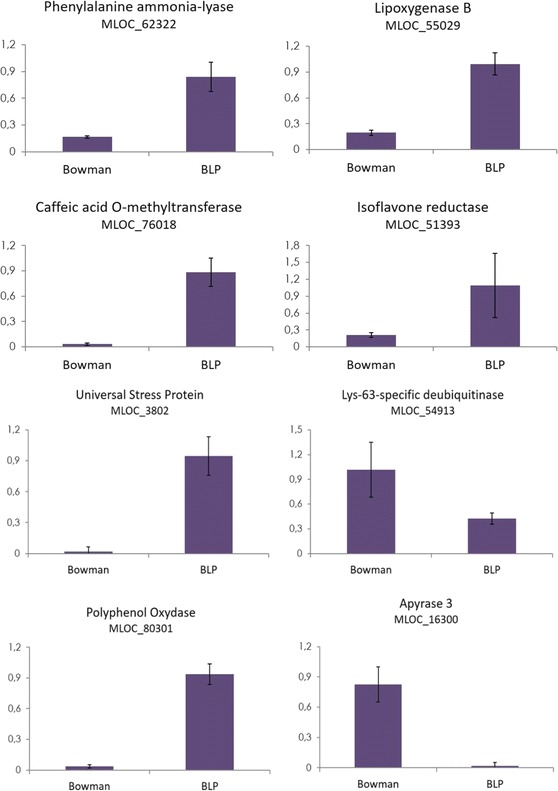
qRT-PCR validation of DEGs (relative mRNA levels of 8 genes obtained using gene-specific primers and cDNA of pericarp and lemma of BLP and BW lines)

qRT-PCR was used to evaluate the transcription of eight genes in seeds of the BLP and Bowman NILs, and results are summarized in Fig. 3. Six genes with higher expression levels in BLP and two genes with higher expression levels in the Bowman line were tested to verify the RNA-seq results.

Among the genes from the RNA-seq data showing increased expression levels in BLP are MLOC_62322 encoding phenylalanine ammonia-lyase (PAL), MLOC_76018 encoding Caffeic acid O-methyltransferase, MLOC_55029 encoding lipoxygenase B, MLOC_51393 encoding isoflavone reductase, MLOC_3802 encoding universal stress protein and MLOC_80301 encoding polyphenol oxidase. We also experimentally verified two genes from the RNA-seq data showing increased expression in the Bowman line: MLOC_54913 encoding lys-63-specific deubiquitinase and MLOC_16300 encoding apyrase 3. The log_2_(FC) values, *p*-values from RNA-seq data corrected with the Benjamini-Hochberg procedure and the qRT-PCR experimental fold changes and associated *p*-values for verified genes are shown in Table 2. The obtained experimental data are consistent with the RNA-seq predictions.

**Table 2:** The log_2_ (FC) values and corrected with Benjamini-Hochberg procedure *p*-value predicted by RNA-seq data and experimental fold changes and *p*-value for verified genes

Gene	Product	log_2_(FC)	*p*-value (RNA-seq)	Experimental expression fold change	*p*-value (experimental)
MLOC_62322	Phenylalanine ammonia-lyase	-3,97	1,1·10^−7^	5,05	0,0095
MLOC_76018	Caffeic acid O-methyltransferase	−8,81	2·10^−40^	27,31	0,0059
MLOC_55029	Lipoxygenase B	−4,14	2,4·10^−21^	5,06	0,0031
MLOC_51393	Isoflavone reductase	−10	1,9·10^−4^	5,15	0,0578
MLOC_3802	Universal Stress Protein	−6,46	6,8·10^−39^	45,08	0,0068
MLOC_80301	Polyphenol Oxydase	−4,61	8,2·10^−31^	26,07	0,0018
MLOC_54913	Lys-63-specific deubiquitinase	6,45	4,5·10^−2^	2,39	0,0423
MLOC_16300	Apyrase 3	11,95	2,4·10^−14^	43,33	0,0064

## Discussion

The diversity of metabolic pathways up- and down-regulated in the presence of *Blp* (*Blp1*) suggests a pleiotropic nature of this gene. This finding is in agreement with previous observations at the phenotypic and physiological levels, suggesting that in addition to conferring black lemma and pericarp colour, the presence of the dominant *Blp* allele may contribute to total antioxidant level in barley grains (Additional file 6), abiotic stress tolerance [11–13] and disease resistance [14–16]. Investigation of DEG functions allowed us to identify genes and metabolic pathways presumably related to known phenotypic and physiological effects of the *Blp* locus.

Black ‘melanin-like’ pigments in plants have complex structures and can withstand almost all solvents of different chemical natures [4]. It has been proposed that these pigments are high molecular weight products of oxidation and polymerization of phenolic compounds [3, 21]. Indeed, an increase of total phenolic compounds in BLP in comparison to BW has been observed [20]. Here, we report significantly increased levels of *PAL* mRNA in the BLP line (Table 2, Fig. 3). PAL encodes phenylalanine ammonia-lyase, the key enzyme of the phenylpropanoid pathway, underlying biosynthesis of phenolic compounds in plants. Nevertheless, these quantitative changes in phenolic metabolites and *PAL* transcripts do not explain the qualitative differences between genotypes carrying dominant and recessive *Blp* alleles. Investigating additional DEGs, we propose that black pigmentation may be associated with high expression level of polyphenol oxidase (PPO) in BLP (Table 2, Fig. 3). PPO catalyses the oxidation of phenols to quinones. The products of the polymerization of quinones cause the darkening of some vegetables and fruits upon contact with air [22]. In humans, a similar enzyme, tyrosinase, is involved in the synthesis of melanin [23].

In wheat, black-spiked genotypes carry dominant allele *Bg* (*Rg-A1c*) on chromosome 1A, however on the short arm [24, 25], so barley *Blp* and wheat *Bg* are unlikely to be orthologues. The origin of black glume colour in wheat remains unknown, although it has been suggested to be related to phlobaphenes, a class of flavonoid compounds [26]. In barley, however, we did not observe phlobaphene biosynthesis structural genes among the identified DEGs.

Higher resistance of black grains to oxidative stress may also associated with increased phenolic content, since many phenolic compounds are known to be antioxidants [27]. Among the DEGs was a gene related to phenolic metabolism, which is of particular interest in relation to mechanisms increasing the antioxidant capacity of grains in the BLP line. This gene, with increased mRNA level in BLP, encodes Caffeic acid O-methyltransferase (Table 2, Fig. 3), which converts caffeic acid into ferulic acid [28]. Ferulic acid is a ubiquitous plant constituent that arises from the metabolism of phenylalanine and tyrosine. Ferulic acid, which accumulates primarily in seeds and leaves (both in its free form as well as covalently linked to lignin or other biopolymers), is known for its ability to terminate free radical chain reactions and hence serves an important antioxidant function to preserve the physiological integrity of cells in unfavourable conditions [29].

Higher resistance of black grains to oxidative stress may partially explain earlier findings that black seeded types are more drought tolerant, more vigorous in early stages of growth and more cold-tolerant than white seeded types [11]. Higher drought tolerance may also be related to cuticular wax biosynthesis, as revealed in our study as one of the metabolic pathways induced in the presence of *Blp*.

Antioxidant capacity can contribute to higher biotic stress resistance.

Black grain colour is associated with higher resistance to *Fusarium* in barley [14–16] and oat [17]. Fusarium head blight is a fungal disease favoured by humid conditions during flowering and early stages of kernel development. Due to better heat accumulation and accelerated drying, black-grained cereals may have an advantage. In addition to this possible effect of black pigmentation, the elevated resistance of dominant *Blp* allele carriers to *Fusarium graminearum* may also be explained by intensive production of phenolic compounds. Gunnaiah et al. applied an integrated metabolo-proteomic approach to decipher the mechanisms by which a wheat QTL (*Fhb1*) contributes to resistance against *F. graminearum* [30]. *Fhb1* is not related to *Blp* or black grain pigmentation. However, it confers resistance to *F. graminearum* by inducing the phenylpropanoid pathway, leading to secondary cell wall thickening in rachises of wheat NILs with a resistant *Fhb1* allele. Finally, increased transcriptional activity of the *PAL* gene can affect the biosynthesis of suberin and phytoalexins. Suberin, a waterproof waxy substance found in higher plants, can benefit black-grained cereals in humid conditions by preventing *F. graminearum* development, while phytoalexins (a wide class of plant molecules known for their antimicrobial properties) can act as toxins for the fungi. In addition to the *PAL* gene, we identified another DEG important for phenolic phytoalexin synthesis, encoding Isoflavone reductase (IFR) (Table 2, Fig. 3). Furthermore, DEGs necessary for the synthesis of terpenoid phytoalexins were identified (Additional file 2; ‘diterpene phytoalexins precursors biosynthesis’).

Improved resistance to *F. graminearum* in *Blp* carriers can also be explained by cuticular wax formation. Cuticular wax biosynthesis was revealed in our study as a metabolic pathway induced in the presence of *Blp*. Another mechanism of resistance can putatively be related to increased expression of the gene encoding universal stress protein (USP), which is usually expressed under environmental stress and stimulates further stress tolerance [31]. The BLP line has an increased USP level in optimal conditions, so it may potentially have increased tolerance to environmental stresses even in the absence of preliminary stimulation.

## Conclusions

Metabolic pathways and genes presumably associated with black lemma and pericarp colour as well as with the pleiotropic effects of the *Blp* locus (i.e., resistance to oxidative stress and pathogens) were revealed. We suggest that black pigmentation of lemmas and pericarps may be related to increased level of phenolic compounds and their oxidation. The effect of *Blp* presence on the synthesis of ferulic acid and other phenolic compounds may be one of the explanations for the increased antioxidant capacity and biotic and abiotic stress tolerance of black-grained cereals. Their drought tolerance and resistance to diseases affecting the spike may also be related to cuticular wax biosynthesis. In addition, upregulated synthesis of phytoalexins, suberin and universal stress protein (USP) in lemmas and pericarps of the *Blp* carriers may also contribute to their increased disease resistance. Further description of the DEGs haplotypes and study of their association with physiological characteristics may be useful for future application in barley pre-breeding.

## Methods

### Plant materials, RNA extraction, genotyping and TAC measurements

Two *H. vulgare* cultivar Bowman [32] near-isogenic lines (NILs) were used in the study: ‘Black lemma and pericarp 1.b’, BLP (NGB20470) with black lemma and pericarp, and ‘Bowman From Fargo’, BW (NGB22812) with green lemma and uncoloured pericarp. These lines were kindly provided by the Nordic Gene Bank (NGB, www.nordgen.org). Plants were grown at the ICG Greenhouse Core Facilities (Novosibirsk, Russia) under 12 h of light per day at 20–25 °C. Plants were genotyped and used for RNA-seq analysis. DNA was extracted for genotyping with microsatellite markers from leaf material (during leaf development stage, BBCH code 12) using a procedure described by Plaschke et al. [33]. For amplification of the barley 1H microsatellite loci, primers [34, 35] were used in PCRs conducted according to Röder et al. [36]. The resulting amplicons were visualized after separation through a 5% agarose (ACTGene, Inc., Piscataway, NJ, USA) gel. The scheme of donor fragments in chromosome 1H of BLP NIL is presented in Additional file 7.

Total RNA was extracted from lemma and pericarp tissue (early dough stage maturity, BBCH code 83) from BLP and Bowman lines using an RNeasy Plant Mini Kit (QIAGEN, Hilden, Germany). RNA extracted from several plants was pooled to exclude possible errors introduced by deviations of biological material. Three biological replicates were prepared for each genotype.

Extracts for TAC measurements were obtained by incubating 1 g of whole grain powder in either 10 ml of 1% HCl for 1 h at 37 °C (method 1) or 10 ml of 1% HCl in 40% ethanol for 30 min at 37 °C (method 2). Three replicates for each line were analysed in case of each methods). Measurements were performed with the antioxidant activity analyser ‘Blizar’ (Interlab Ltd., Russia) according to the manufacturer’s instructions. Results are presented in Additional file 6.

### RNA preparation and sequencing

RNA was pooled into six libraries (three replicates for each line). ERCC spike-in mix was added to each library to serve as an internal control. Libraries were incubated with poly-T-tailed beads for poly-A enrichment. Library RNA was fragmented by incubation with nuclease enzymes. Sequencing was carried out with the IonTorrent platform.

### Read preprocessing and mapping

Prinseq [37] tool v 0.20.4 was used to assess sequence quality and filter the libraries. Sequences with length below 50 nucleotides and mean Phred quality less than 20 were excluded from further analysis. Filtered libraries were mapped to *H. vulgare* genome assembly version 32,608 v 1.33 from the Ensembl Plants database (http://plants.ensembl.org). Mapping was performed with TopHat2 tool v 2.1.0 [38] after constructing genome indexes using Bowtie2 v 2.2.6 software [39]. TopHat2 parameters ‘allowed mismatches’, ‘read edit distance’ and ‘read gap length’ were set at 4.

After mapping libraries to the reference genome, the resulting alignments were processed with the Cufflinks v 2.2.1 pipeline [40]. The number of reads mapped to each genome fragment, either expressed or annotated in the genome assembly (referred to here as ‘transcripts’), and respective RPKM (Reads Per Kilobase per Million mapped reads) [41] values were then used to detect differential expression of genes between the two studied barley lines. Furthermore, dendrogram of library clustering was plotted using the cummeRbund [42] package for R. Whole list of transcripts identified by cufflinks pipeline was used as a subset.

### Gene expression analysis

Differential expression search was carried out using the Cufflinks pipeline cuffdiff utility and EdgeR v 3.8.6 [43] package for R v 3.1.3. Likelihood ratio tests were performed with EdgeR. Transcripts with total RPKM values below 15 in three libraries belonging to either of the biological lines were discarded from analysis. Transcripts with two-fold and higher differences in expression levels (|logFC| > 1) and FDR < 0.05 for EdgeR or q < 0.05 for Cufflinks were considered differentially expressed between the two lines. Transcripts with higher and lower levels of expression in BLP were analysed separately.

### Transcript functional annotation

Blastp utility from the BLAST suite v 2.6.0 [44] was used to align sequenced of differentially expressed transcripts to sequences of the barley Affymetrix genome array downloaded from the AgriGO database [45]. For each transcript, the sequence from the barley Affymetrix genome array with the highest homology was identified. Lists of Affymetrix sequences corresponding to the transcripts with heightened and lowered levels of expression in BLP were processed with the AgriGO database Singular Enrichment Analysis tool.

Annotated genes with differential expression were analysed using the Plant Metabolic Network (PMN) database, BarleyCyc division (http://plantcyc.org/databases/barleycyc/5.0).

### qRT-PCR

For qPCR, isolated RNA was treated with DNase (QIAGEN RNase-Free DNase Set). Then, 0.7 μg of RNA was used to prepare single-stranded cDNA by reverse transcription, based on a RevertAid™ kit (Thermo Fisher Scientific Inc., Waltham, MA, USA) and a (dT)_15_ primer.

Primers were designed using IDT PrimerQuest software (http://eu.idtdna.com/PrimerQuest/Home/) for eight DEGs (Table 3).

**Table 3:** Primers sequences constructed and used in qRT-PCRs

Gene	Product	Primers sequences
MLOC_16300	Apyrase 3	Forward 5’CCTATGGGTTGCTCTGAATTAC3’, Reverse 5’AGTAGTCTCCTCCCAGTTTAC3’
MLOC_51393	Isoflavone reductase	Forward 5’GCTTTCTTCCCGCATTCT3’, Reverse 5’GATACGATGGCAGATGAACTAA3’
MLOC_55029	Lipoxygenase B	Forward 5’ACAGAAACGGACCCTAGAT3’, Reverse 5’CCTGGTTATCGGAAGAATGG3’
MLOC_76018	Caffeic acid O-methyltransferase	Forward 5’CAAGAAGTACCCGAGCATAAA3’, Reverse 5’CGGCACCATCTCAAACAT3’
MLOC_62322	Phenylalanine ammonia-lyase	Forward 5’GCTCATGTTTGCCCAATTC3’, Reverse 5’GGAAAGGTTGGAAGGTAGAC3
MLOC_80301	Polyphenol Oxydase	Forward 5’AGCAAGGAGAAGGAGGAG3’, Reverse 5’GCACGTCGAACTTGATGA3’
MLOC_3802	Universal Stress Protein	Forward 5’CCGGAGGTGATGAAGAACTA3’, Reverse 5’ATGACGATGGTGTCGATCT3’
MLOC_54913	Lys-63-specific deubiquitinase	Forward 5’CGGCAAGACATCTACCAATAG3’, Reverse 5’GCACTGTTATCCGGTTCATAA3’

The *Ubc* (ubiquitin) gene sequence was used as a reference [46]. The subsequent qRT-PCR was based on a SYNTOL SYBR Green I kit (Syntol, Moscow, Russia). Three technical replicates of each reaction were performed.

## Additional files

Additional file 1: Tables “Genes” (List of genome fragments identified by TopHat2, their genomic location, RPKM values in six samples and annotation), “Cufflinks” (Differentially expressed genes identified by Cufflinks), “EdgeR” (Differentially expressed genes identified by EdgeR), Table “UpRegDEGS_Cufflinks & EdgeR” (Differentially expressed genes identified by Cufflinks and EdgeR, upregulated in BLP line), and “DownRegDEGS_Cufflinks & EdgeR” (Differentially expressed genes identified by Cufflinks and EdgeR, downregulated in BLP line). (XLSX 2557 kb)

Additional file 2: Links of differentially expressed genes identified by Cufflinks and EdgeR programs to BarleyCyc pathways. (XLSX 25 kb)

Additional file 3: Ontology terms associated with biological process of protein products for genes with higher expression level in BLP line. (PNG 130 kb)

Additional file 4: Ontology terms associated with cellular localization of protein products for genes with lower expression level in BLP line. (PNG 189 kb)

Additional file 5: Ontology terms associated with molecular functions of genes with lower expression level in BLP line. (PNG 37 kb)

Additional file 6: The total antioxidant content (mg/g) in grains of Bowman (BW) and BLP lines (equivalent to Gallic acid). Method 1: extracts were prepared by incubation of 1 g of whole grain powder in 10 ml of 1% HCl for 1 h at 37 °C. Method 2: 1 g of whole grain powder was incubated in 10 ml of 1% HCl in 40% ethanol for 30 min at 37 °C. Measurements were performed with the antioxidant activity analyser ‘Blizar’ (Interlab Ltd., Russia) according to the manufacturer’s instructions. * - difference between BW and BLP is significant (*p* ≤ 0.01; *U*-test). (PDF 183 kb)

Additional file 7: Chromosome 1H scheme of Bowman (BW) and BLP lines. *Blp* donor segment on chromosome 1H remaining in the BLP NIL, revealed by microsatellite genotyping, is in gray. (PDF 49 kb)

## Abbreviations

BLP: Black lemma and pericarp
BW: Bowman
DEGs: Differently expressed genes
FHB: Fusarium head blight
GO: Gene ontology
IFR: Isoflavone reductase
PAL: Phenylalanine ammonia-lyase
PPO: Polyphenol oxidase
PPS: Pathway Perturbation Scores
qRT-PCR: Quantitative RT-PCR
RPKM: Rids Per Kilobase per Million of reads
RT: Reverse transcription
TAC: The total antioxidant content
