# Supplementary figures and images for: Metabolic pathways and genes identified by RNA-seq analysis of barley near-isogenic lines differing by allelic state of the Black lemma and pericarp (Blp) gene
Source: BMC Plant Biol. 2017 Nov 14;17(Suppl 1):182. doi: 10.1186/s12870-017-1124-1 (PMC5688459; doi:10.1186/s12870-017-1124-1)

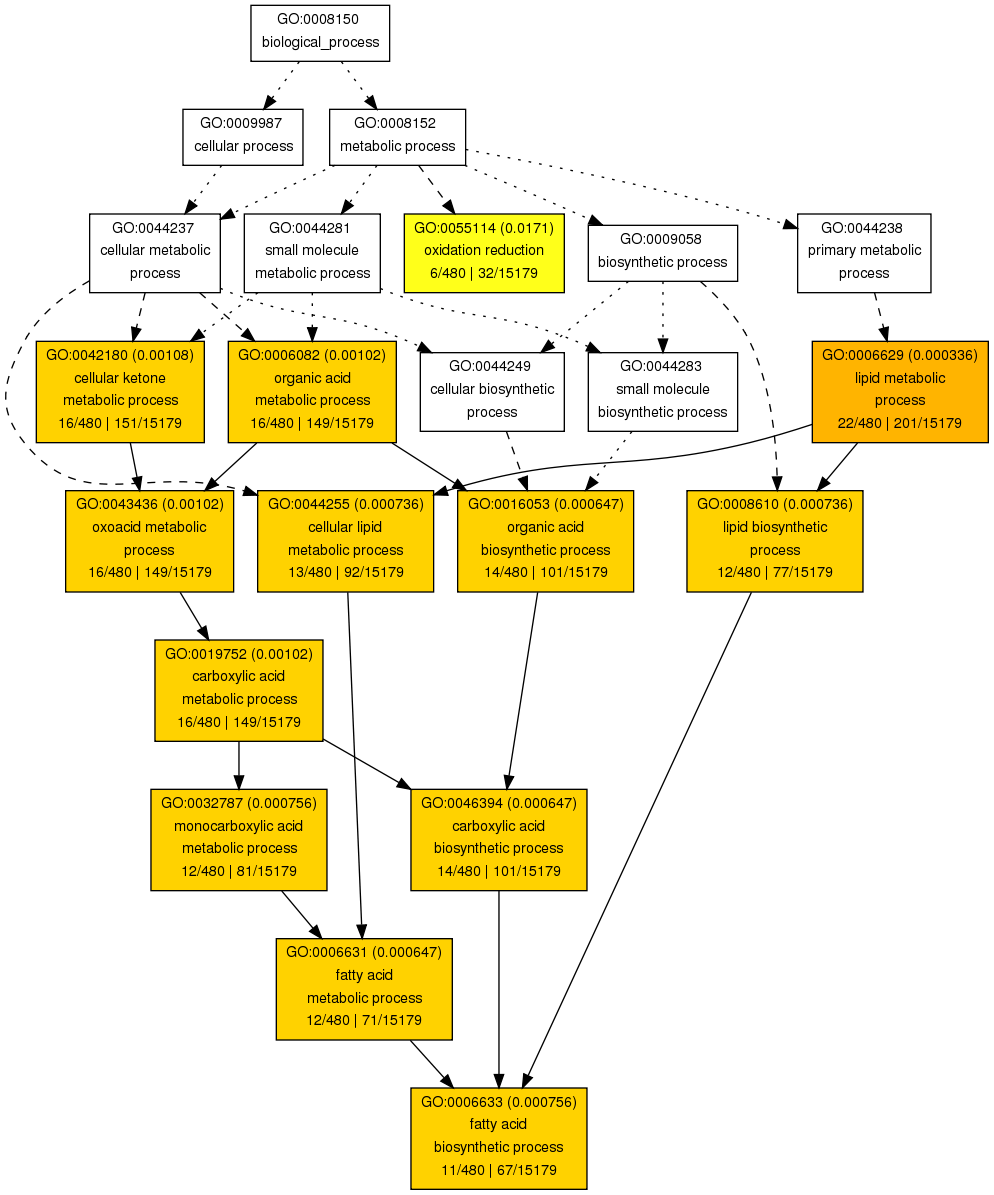

Supplement: Supplementary file 3 — Ontology terms associated with biological process of protein products for genes with higher expression level in BLP line. (PNG 130 kb) [file 12870_2017_1124_MOESM3_ESM.png]

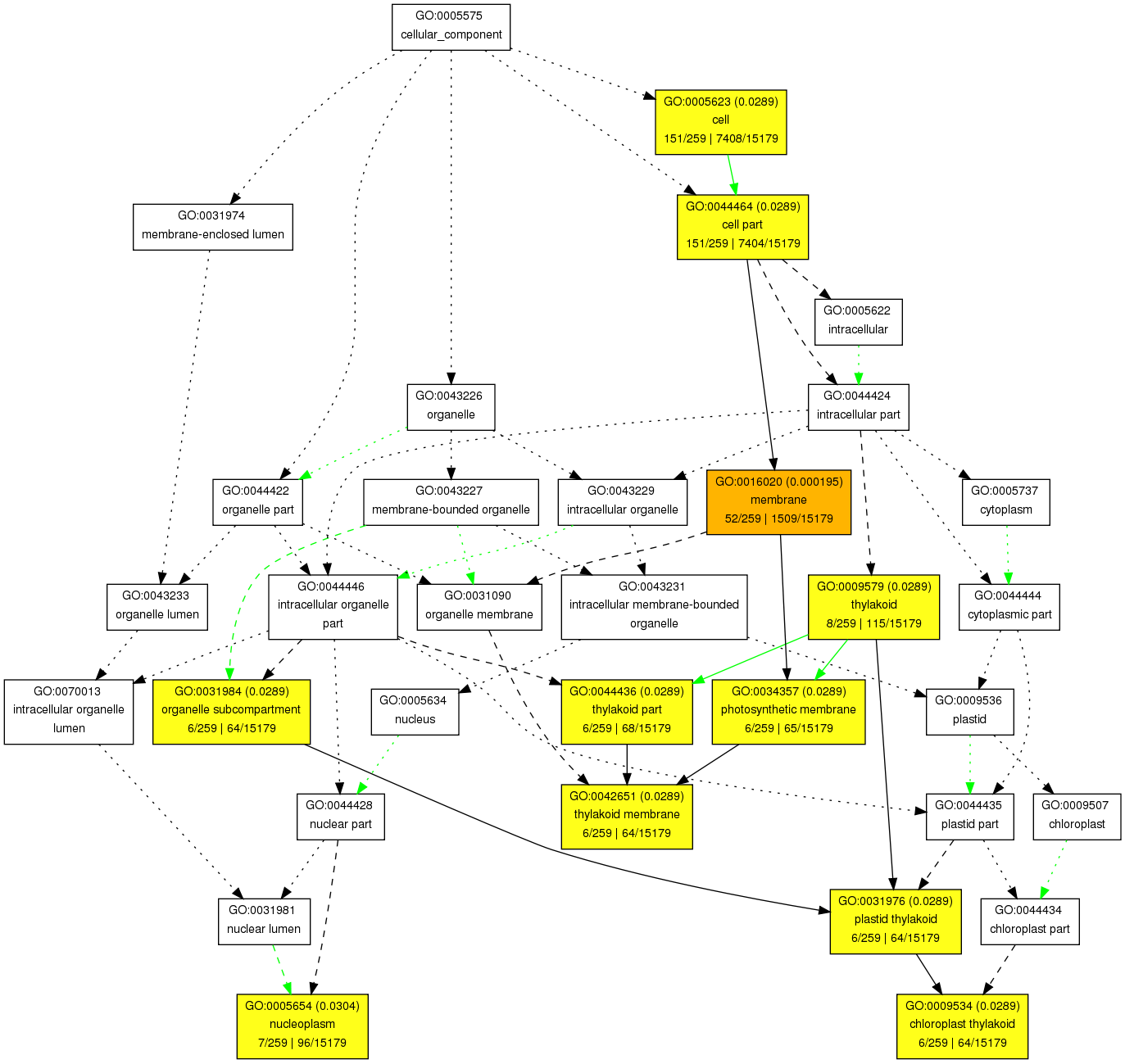

Supplement: Supplementary file 4 — Ontology terms associated with cellular localization of protein products for genes with lower expression level in BLP line. (PNG 189 kb) [file 12870_2017_1124_MOESM4_ESM.png]

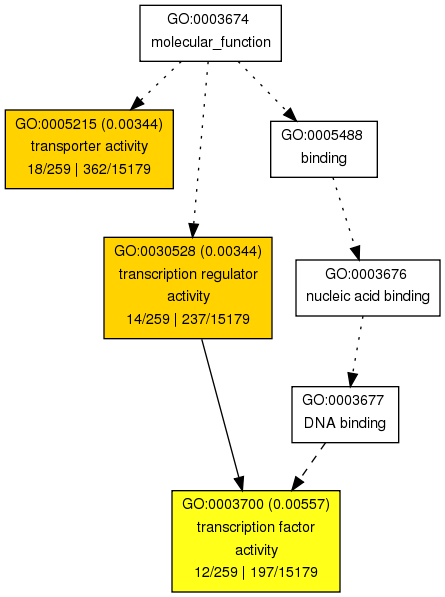

Supplement: Supplementary file 5 — Ontology terms associated with molecular functions of genes with lower expression level in BLP line. (PNG 37 kb) [file 12870_2017_1124_MOESM5_ESM.png]

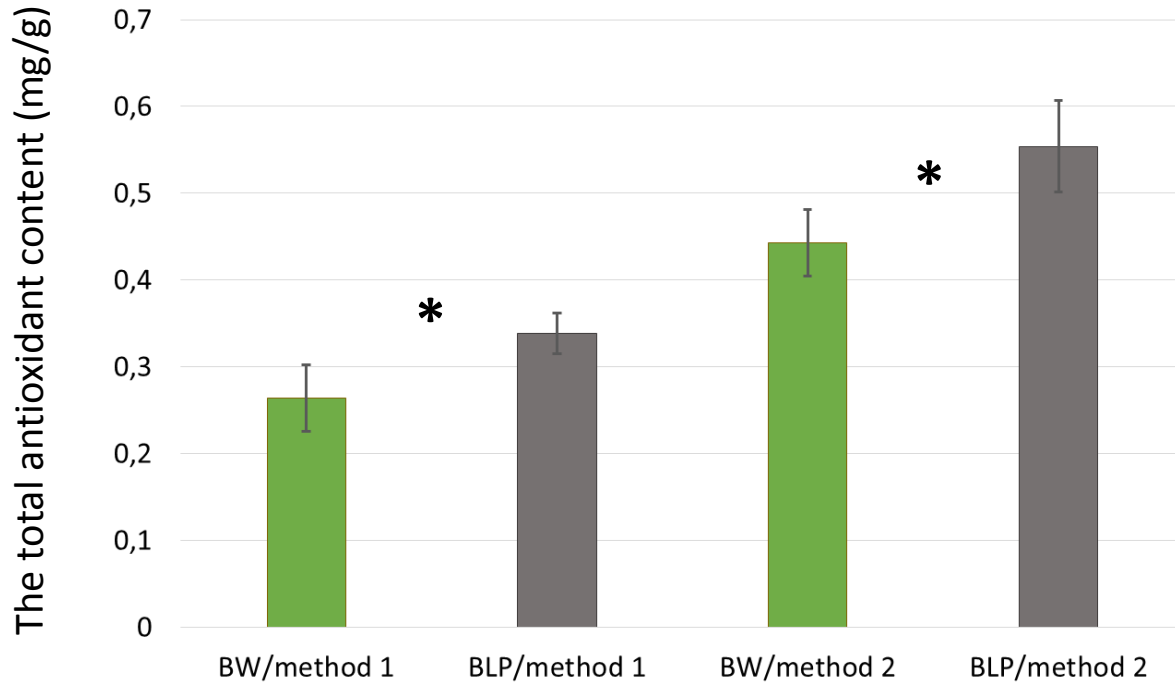

Supplement: Supplementary file 6 — The total antioxidant content (mg/g) in grains of Bowman (BW) and BLP lines (equivalent to Gallic acid). Method 1: extracts were prepared by incubation of 1 g of whole grain powder in 10 ml of 1% HCl for 1 h at 37 °C. Method 2: 1 g of whole grain powder was incubated in 10 ml of 1% HCl in 40% ethanol for 30 min at 37 °C. Measurements were performed with the antioxidant activity analyser ‘Blizar’ (Interlab Ltd., Russia) according to the manufacturer’s instructions. * - difference between BW and BLP is significant (p ≤ 0.01; U-test). (PDF 183 kb) [file 12870_2017_1124_MOESM6_ESM.pdf]

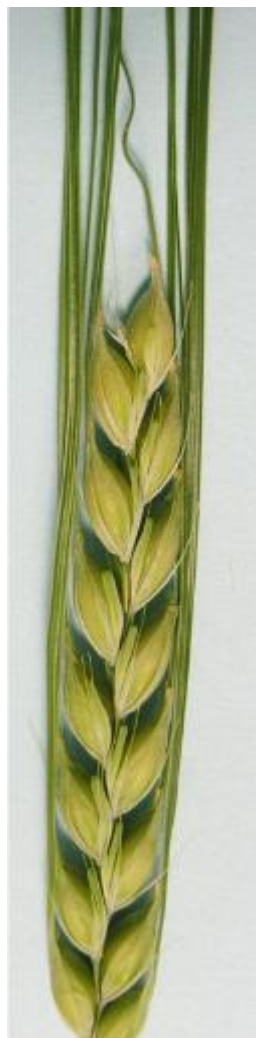

Bowman  
(NGB22812)

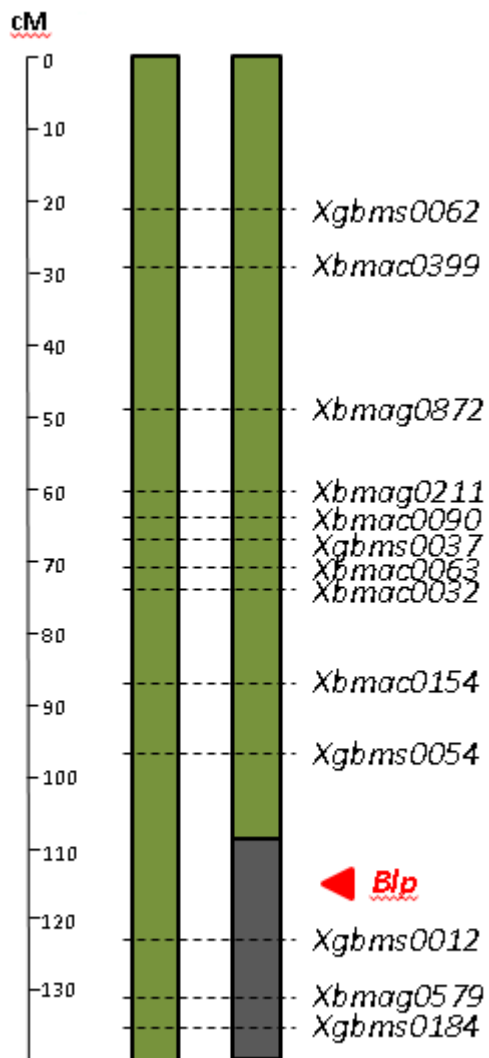

chromosome 1H

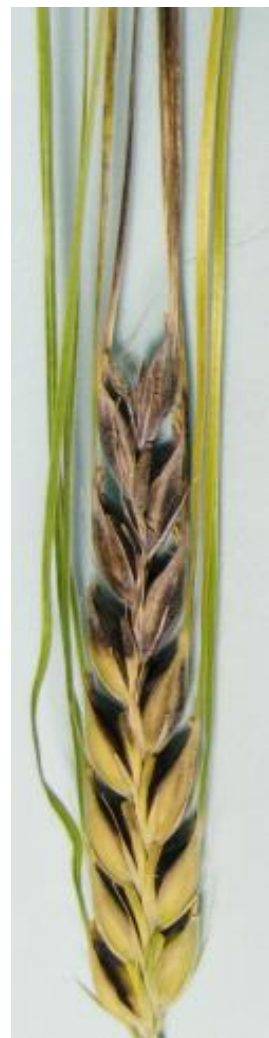

BLP  
(NGB20470)

Supplement: Supplementary file 7 — Chromosome 1H scheme of Bowman (BW) and BLP lines. Blp donor segment on chromosome 1H remaining in the BLP NIL, revealed by microsatellite genotyping, is in gray. (PDF 49 kb) [file 12870_2017_1124_MOESM7_ESM.pdf]
